# Characteristics of traumatic brain injury patients with abnormal neuroimaging in Southeast Norway

**DOI:** 10.1186/s40621-020-00269-8

**Published:** 2020-09-01

**Authors:** Cathrine Tverdal, Mads Aarhus, Nada Andelic, Ola Skaansar, Karoline Skogen, Eirik Helseth

**Affiliations:** 1 Department of Neurosurgery, Ullevål Hospital, Oslo University Hospital, P. O. Box 4956 Nydalen, N-0424 Oslo, Norway; 2 Institute of Clinical Medicine, Faculty of Medicine, University of Oslo, Oslo, Norway; 3 Department of Physical Medicine and Rehabilitation, Oslo University Hospital, Oslo, Norway; 4 Institute of Health and Society, Research Centre for Habilitation and Rehabilitation Models and Services (CHARM), Faculty of Medicine, University of Oslo, Oslo, Norway; 5 Department of Neuroradiology, Oslo University Hospital, Oslo, Norway

**Keywords:** Traumatic brain injury, Neurosurgery, Trauma center, Hospital admission

## Abstract

**Background:**

The vast majority of hospital admitted patients with traumatic brain injury (TBI) will have intracranial injury identified by neuroimaging, requiring qualified staff and hospital beds. Moreover, increased pressure in health care services is expected because of an aging population. Thus, a regular evaluation of characteristics of hospital admitted patients with TBI is needed. Oslo TBI Registry – Neurosurgery prospectively register all patients with TBI identified by neuroimaging admitted to a trauma center for southeast part of Norway. The purpose of this study is to describe this patient population with respect to case load, time of admission, age, comorbidity, injury mechanism, injury characteristics, length of stay, and 30-days survival.

**Methods:**

Data for 5 years was extracted from Oslo TBI Registry – Neurosurgery. Case load, time of admission, age, sex, comorbidity, injury mechanism, injury characteristics, length of stay, and 30-days survival was compiled and compared.

**Results:**

From January 1st, 2015 to December 31st, 2019, 2153 consecutive patients with TBI identified by neuroimaging were registered. The admission rate of TBI of all severities has been stable year-round since 2015. Mean age was 52 years (standard deviation 25, range 0–99), and 68% were males. Comorbidities were common; 28% with pre-injury ASA score of ≥3 and 25% used antithrombotic medication. The dominating cause of injury in all ages was falls (55%) but increased with age. Upon admission, the head injury was classified as mild TBI in 46%, moderate in 28%, and severe (Glasgow coma score ≤ 8) in 26%. Case load was stable without seasonal variation. Majority of patients (68%) were admitted during evening, night or weekend. 68% was admitted to intensive care unit. Length of hospital stay was 4 days (median, interquartile range 3–9). 30-day survival for mild, moderate and severe TBI was 98, 94 and 69%, respectively.

**Conclusions:**

The typical TBI patients admitted to hospital with abnormal neuroimaging were aged 50–79 years, often with significant comorbidity, and admitted outside ordinary working hours. This suggests the necessity for all-hour presence of competent health care professionals.

## Background

Traumatic Brain Injury (TBI) is globally recognized as a major health and socioeconomic issue (James et al. 2019). TBI is defined as alteration in brain function, or other evidence of brain pathology, caused by an external force (Menon et al. 2010). The definition is wide, and the term TBI represents a heterogeneous group of patients.

The international incidence of TBI in a general population is estimated as high as 369–790/100000 (James et al. 2019; Feigin et al. 2013), the vast majority being minimal and mild TBIs. Narrowing down to hospital admitted TBI, the incidence rate in high-income countries drops to 83–262/100000, with increasing fractions of moderate and severe TBIs (Peeters et al. 2015; Pedersen et al. 2015; Koskinen and Alaranta 2008; Rickels et al. 2010; Heskestad et al. 2009; Andelic et al. 2008; Centers for Disease Control and Prevention 2019). Refining it even further to hospital admitted patients with abnormal traumatic intracranial findings on computed tomography (CT), the incidence rate in Scandinavian countries drops to 26–42/100000 (Pedersen et al. 2015; Heskestad et al. 2009; Andelic et al. 2008).

If head-injured patients are triaged according to established guidelines, the vast majority admitted to hospital will have TBI identified by neuroimaging (Sollid et al. 2008; Unden et al. 2013; National Institute for Health and Care Excellence 2014; Pandor et al. 2011). With guideline compliance, these patients will represent the majority of hospital admitted TBI patients in present and near future.

**Fig. 1:**
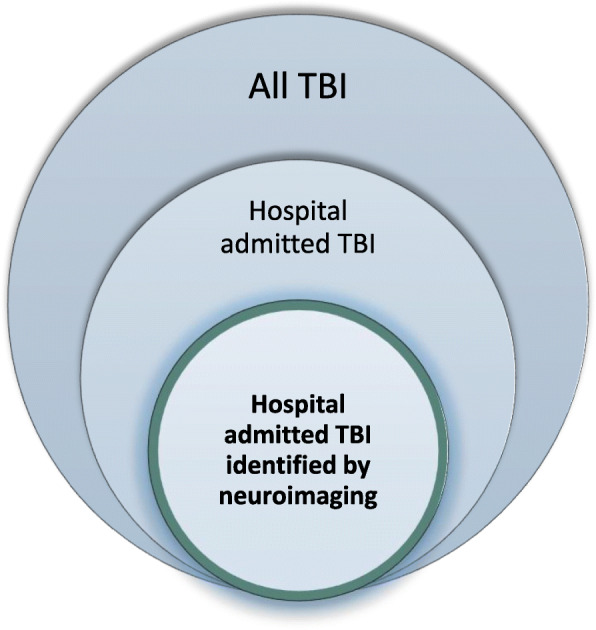
Illustration of the patient population with TBI included in the Oslo TBI Registry – Neurosurgery. The patient population with TBI in the South East health region, only patients with verified TBI on neuroimaging and admitted to OUH are included in the Oslo TBI Registry – Neurosurgery

To describe this group of TBI patients, the Department of Neurosurgery at Oslo University Hospital (OUH) established in 2015 the Oslo TBI Registry – Neurosurgery for admitted patients with TBI identified by neuroimaging (Fig. 1). The registry includes information about patient characteristics, injury mechanism, neuroimaging findings, neurosurgical interventions, length of stay, discharge status, post discharge destination, and 30-day survival. The overall registry’s purpose is to optimize patient management and adequately delegate the use of hospital resource.

In trauma care organizations, hospital resources include adequate and qualified staff, staffed operating theaters, access to imaging, available beds and support services. Hence, for in hospital resource planning accurate information on patient load, patient characteristics, injury severity and time of admittance is of great importance. Several studies have shown the number of admissions for trauma patients increases on weekends, in the evenings, and are subject to seasonal variation (Kieffer et al. 2016; Robertson and Giannoudis 2004; Stonko et al. 2018; Vaziri et al. 2007; Ali and Willett 2015; Rising et al. 2006; Roislien et al. 2018). We wanted to study the extent this is applicable to TBI patients. The study may be of international interest as Norway has a well-organized public trauma system with a highly developed infrastructure and transportation systems enabling TBI patients to be transported to the trauma centers as well as well-equipped trauma teams in centers for definitive treatment of patients (Sollid et al. 2008).

The aim for this study was to describe this patient population with respect to case load, time of admission, seasonal variations, age, sex, comorbidity, injury mechanism, type and severity of TBI, length of hospital stay and 30-day survival.

## Methods

### Study design

A descriptive study of TBIs, identified by neuroimaging, admitted to Oslo University Hospital (OUH) in the time period January 1st, 2015 to December 31st, 2019.

### Study setting

Norway has a population of 5.3 million, the mainland covers 323,781 km^2^, and is divided into four geographical healthcare regions. The South-East region is the largest with a population of 3,032,670 in 2019 (Statistics Norway n.d.) and covers 110,000 km^2^ with urban and rural areas. The region encompasses 19 local hospitals that provide acute care and general surgical assessment, management and stabilization (Helse og omsorgsdepartementet 2017). Oslo University Hospital (OUH) is the only Level I trauma center with neurosurgical services in the region. OUH also serves as the primary trauma referral hospital for Oslo, with a population of 693,491 in 2019 (Statistics Norway n.d.). Norway provides universal healthcare to all Norwegian residents.

Trauma patients with severe injuries or suspected severe TBI are transported directly and admitted to OUH. The remaining trauma patients are admitted to local hospitals for management and only transferred to OUH if deemed necessary. Treatment of severe TBI at OUH follows the Brain Trauma Foundation guideline (Ronning et al. 2018; Sovik et al. 2014; Brain Trauma Foundation 2016). Minimal, mild and moderate TBI patients are managed according to the Scandinavian guidelines for initial management of minimal, mild and moderate head injuries in adults and in children (Astrand et al. 2016; Unden et al. 2013).

### Database

The Oslo TBI Registry – Neurosurgery is a quality control database run by the neurosurgical department at OUH since January 1st, 2015. The Neurosurgeons registered patients prospectively on daily basis and a dedicated group collected data for each patient (CT, OS, EH). Data is retrieved from electronic medical records and stored in Medinsight (version 2.12.4.0), a database approved by the OUH data protection officer. Medinsight is linked to the National Population Register and date of death is automatically updated. Inclusion criteria are: (a) traumatic brain injury; (b) cerebral-CT/CTA or cerebral-MRI/MRA with findings of acute trauma (hemorrhage, fracture, traumatic axonal injury, vascular injury); (c) admitted to OUH within 7 days post-injury; (d) having a Norwegian social security number. Data for this study was retrieved from the Oslo TBI Registry – Neurosurgery on February 17th, 2020.

### Variables

#### Demographic factors

Age was retrieved as a continuous variable and categorized into five-year intervals. Sex was registered as male or female. Living status at time of injury is registered into home – independent, home – with assistance, or institutionalized.

#### Comorbidity

Pre-injury health status was assessed using three different categories: (i) American Society of Anesthesiologists ASA Physical Status Classification System (ASA1: normal healthy; ASA2: mild systemic disease; ASA3: severe systemic disease; ASA4: severe systemic disease that is a constant threat to life) (American Society of Anesthesiologists 2014); (ii) use of anticoagulation/antiplatelet medication (none, antiplatelet, anticoagulation, both antiplatelet and anticoagulation); (iii) prior substance dependence previously documented in medical records (yes/no).

#### Trauma mechanism

Trauma mechanism was grouped into (i) falls; (ii) motor vehicle crashes (MVC) (including motor cycles, ATV and snowmobile); (iii) pedestrian hit by a motor vehicle; (iv) bicycle (including electric bicycles and scooters) (v) sports; (vi) violence; (vii) self-harm; (viii) other or unknown. High energy as yes/no (“yes”; fall from height ≥ 3 m, MVC, bicycle, or when high-energy was described; e.g. downhill skiing). Alcohol influence at time of injury as yes/no (“yes”; either positive blood alcohol level or description of alcohol intake before injury).

#### Neuroimaging

Cerebral-CT was done as part of the primary assessment and an additional cerebral-MRI was only performed on clinical indication. The following traumatic imaging findings were registered (yes/no); cranial fracture, epidural hematoma (EDH), acute subdural hematoma (SDH), traumatic subarachnoid hemorrhage (tSAH), intraventricular hemorrhage (IVH), brain contusion, traumatic axonal injury (TAI), and penetrating injury. The Rotterdam CT score was used to classify CT findings and was assessed on the initial cerebral-CT with a range from 1 to 6 (worst score = 6). The score is based on (i) status of basal cisterns (normal, compressed, or absent); (ii) midline shift (0–5 mm or > 5 mm); (iii) epidural hematoma (present or absent); (iv) traumatic subarachnoid hemorrhage/intraventricular hemorrhage (present or absent). Increased Rotterdam CT score correlates with increased mortality in patients with severe and moderate TBI (Maas et al. 2005).

#### Injury severity

The Glasgow coma score (GCS) recorded was the lowest score documented in the time window between injury and arrival at the OUH emergency room (ER) or intubation. TBI was categorized according to Head Injury Severity Score (HISS) into minimal (GCS 15 and no loss of consciousness or amnesia), mild (GCS 14 or 15 plus amnesia, or brief (< 5 min) loss of consciousness, or impaired alertness or memory), moderate (GCS 9–13 or loss of consciousness ≥5 min or focal neurological deficit) or severe (Severe: GCS ≤ 8) (Stein and Spettell 1995). In the analysis, minimal TBI with traumatic findings on CT was grouped with mild TBI. In the literature, mild TBI with traumatic findings on CT is referred to as complicated mild TBI (Williams et al. 1990; Iverson et al. 2012).

Multiple trauma was defined as injuries to other parts of the body verified by imaging (skeletal fractures or injury to internal organs) and registered with “no”, “yes, conservative treatment” or “yes, surgical treatment”. Skin injuries were not registered.

#### Hospital admission

Time and date of admission to OUH was registered and from what location (i) scene of accident; (ii) local hospital; (iii) Oslo Emergency Department (separate location from the main hospital); (iv) other. The presence of OUH trauma team (yes/no), which consists of specialty trained physicians and nurses in anesthesia and surgery, radiologist, radiographer and bioengineer. Monday to Friday between 07:00 and 17:00 the hospital is staffed to manage elective and emergency patients, and critically ill inpatients. Beyond these hours, the hospital is mainly staffed for emergencies and care of critically ill inpatients.

All patients admitted to an intensive care unit (ICU) were registered as admitted to ICU regardless of medical condition. In addition, patients admitted to an intermediate unit and in need of any organ-supportive interventions/medication or sedatives, or requiring observation > 24 h, was registered as ICU admitted. Length of stay (LOS) was calculated with formula *LOS = date of discharge – date of admission + 1*. Stay at rehabilitation unit was not included in calculation of LOS.

### Statistical analysis

Descriptive statistics summarize the characteristics of patients, injuries and treatment. Categorical data is presented with frequencies and percentages. Continuous variables are presented with mean (standard deviation; SD) or median (interquartile range; IQR) depending on distribution. To compare group differences, we used Pearson χ^2^ test for categorical variables, and independent *t*-test or Mann-Whitney *U* Test for continuous variables. 30-days survival was estimated using Kaplan-Meier and log-rank test for group comparison. Two-sided *p*-values of 0.05 were considered for statistical significance. Data were analyzed using IBM SPSS© version 25.

### Ethical considerations

Anonymized data was retrieved from the Oslo TBI Registry. The Registry was approved by OUH data protection officer (approval number 2016/17569). This study qualifies as a quality control study, hence application to the regional ethical committee was waived (Regional Committees for Medical and Health Research Ethics (REC), 2012). The study was approved by OUH data protection officer (approval number 18/20658).

## Results

During the five-year study period 2153 consecutive patients with TBI identified by neuroimaging were admitted to OUH and included in this study.

**Fig. 2:**
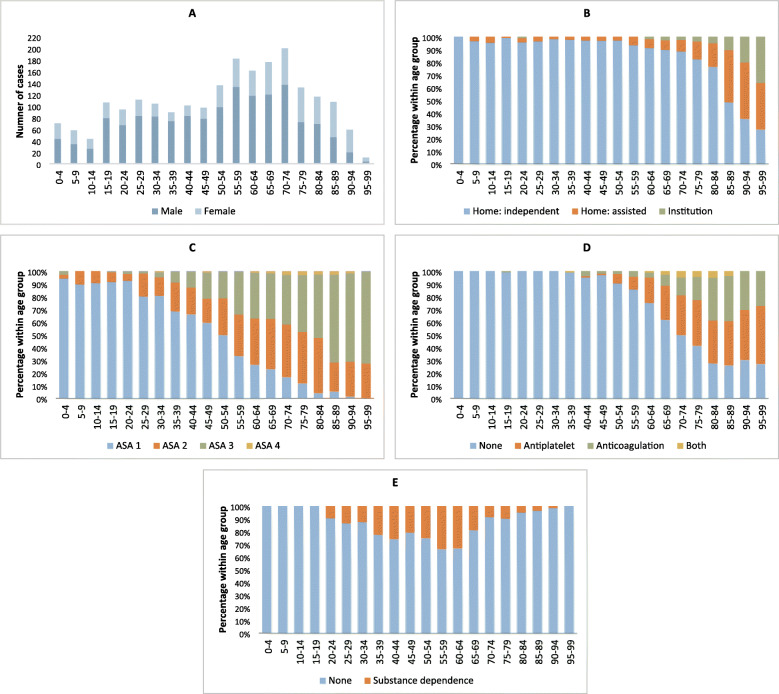
Distribution of age groups and pre-injury status. **a**: Distribution of sex between age groups. **b**: Pre-injury living status. **c**: Pre-injury ASA score **d**: Pre-injury use of antiplatelet and anticoagulation **e**: Pre-injury substance dependence

TBI was seen in all age groups and peaked in the age groups between 55 years and 74 years (Fig. 2a); mean age was 52 years (SD 25, range 0–99). There was a clear male preponderance (68%), especially in younger patients. The male dominance was gradually reduced with increasing age. Median male age was 54 years (mean 50, SD 23), median female age was 63 (mean 57, SD 27) (Mann-Whitney *U* Test, *p* = <.001). The majority lived independently at home (88%) (Table 1, Fig. 2b).

**Table 1:** Comorbidity and injury mechanism

	N (%) 2153 (100)
**Male**	1466 (68)
**Preinjury status**	
ASA 1. Healthy	975 (45)
ASA2. Mild systemic disease	572 (27)
ASA 3. Severe systemic disease	582 (27)
ASA 4. Life-threatening systemic disease	24 (1)
Living home – independent	1883 (88)
Living home - with assistance	192 (9)
Institutionalized	51 (2)
Substance dependency	325 (15)
Antiplatelet	322 (15)
Anticoagulation	189 (9)
Antiplatelet and anticoagulation	34 (1.5)
Influenced by alcohol at time of injury	579 (27)
**Trauma mechanism**	
High-energy trauma (Yes)	812 (38)
Falls	1187 (55)
Motor vehicle crashes	205 (10)
Bicycle	196 (9)
Violence	154 (7)
Sports (except bicycle)	114 (5)
Pedestrian	88 (4)
Self-harm	57 (3)
Other/unknown	152 (7)

Description of comorbidity measured by ASA-PS score, use of antithrombotic medication and substance dependency are given in Table 1 and Figs. 2c-e. In the younger TBI age group the vast majority had an ASA-PS score of 1 or 2. The landscape changed in the elderly group with larger fractions not living independently, and ASA-PS score of 3 or 4 (Fig. 2c). The overall use of antithrombotic medication was 25%, increasing with age with a proportion of 42% in age ≥ 50 (Fig. 2d). Pre-injury substance dependence was registered in 15% and was spread throughout all adult age groups, but most prevalent in middle aged males (Fig. 2e). Alcohol accounted for 9% alone.

**Fig. 3:**
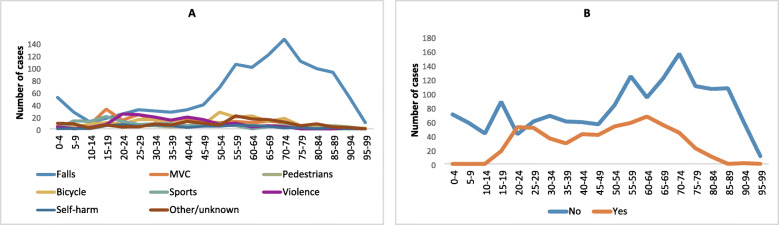
Trauma mechanism and alcohol use. **a**: Distribution of trauma mechanism in age groups. **b**: Distribution of alcohol use before injury in age groups

Trauma mechanism is presented in Table 1 and Fig. 3. Falls were the dominating cause of injury (55%) in all age groups, except patients aged 10–19 years where MVC accounted for 26%, sports 22% and falls 19%. The fraction of fall injuries increased with age, especially in patients’ ≥50 years (Fig. 3a). High-energy trauma was seen in 812/2153 (38%) with a mean age of 43 years (SD 22), in low-energy trauma mean age was 58 years (SD 24) (CI 95% 13–17, *p* = <.001). At time of injury 579/2153 (27%) were under influence of alcohol with a mean age of 49 years (SD 18), patients not influenced by alcohol had mean age 53 years (SD 27) (CI 95% 2.5–6.4, *p* = <.001). The percentage of patients influenced by alcohol was > 30% in age group 20–75 years. No patients < 15 years was influenced by alcohol (Fig. 3b). Males were more often influenced by alcohol (31%) than women (19%) (χ2 (1) =34, *p* = <.001).

Severity of TBI and TBI pathology is presented in Table 2. Median GCS was 13 (IQR 8–15). TBI severity according to HISS was minimal 6%, mild 40%, moderate 28% and severe 26%. When comparing age groups and HISS, mild TBI was more frequent in the youngest and oldest age groups, severe TBI peaked in age group 10–19 years (39%) and gradually decreased with age (χ2 (18) =60, *p* = <.001). There was no difference in HISS between male/female (χ^2^ (2) =4, *p* = .1).

**Table 2:** Severity of TBI and TBI pathology

	N (%) 2153 (100)
**Glasgow coma score**	
14–15	1031 (48)
9–13	571 (27)
6–8	250 (12)
3–5	301 (14)
**Head injury severity score**	
Minimal	129 (6)
Mild	857 (40)
Moderate	612 (28)
Severe	555 (26)
**Rotterdam CT score**	
1–2	849 (39)
3–4	1122 (52)
5–6	182 (9)
Basal cisterns	
Normal	1800 (84)
Compressed/absent	352 (16)
Midline shift	
0–5 mm	1799 (84)
> 5 mm	353 (17)
**Pathology seen on first CT**^**a**^	
Subarachnoid hemorrhage	1235 (57)
Subdural hematoma	1185 (55)
Contusion	1029 (48)
Epidural hematoma	334 (16)
Intraventricular hemorrhage	254 (12)
Skull fracture - basilar	799 (37)
Skull fracture - linear vault	699 (33)
Skull fracture - depressed vault	122 (6)
Penetrating injury	34 (1.6)
**Traumatic Axonal Injury**	
MRI done (Yes)	626 (29)
TAI lesion identified (Yes)	322 (15)

*CT* Computed tomography, *MRI* Magnetic resonance imaging, *TAI* Traumatic Axonal Injury

^a^ One patient may have more than one type of traumatic injury

Rotterdam CT score ≥ 4 was seen in 362/2153 (17%). Compressed/absent basal cisterns were seen in 352 (16%) and midline shift ≥5 mm and 353/2153 (17%). The most frequent pathologies in this series were SDH, tSAH, brain contusion and cranial fracture. Multiple pathologies were seen in the majority (70%), e.g. simultaneous cranial fracture, SDH, tSAH and brain contusion. Higher age was significantly associated with SDH (*p* = <.001), tSAH (*p* = <.001) and cranial fracture (*p* = <.001). Younger age was associated with EDH (*p* = <.001). Penetrating injury rarely occurred (1.6%). MRIs were done in 626/2153 (29%) and accounted for younger patients, mean age 39 (SD 23) compared to those without MRI, mean age 58 years (SD 23) (CI 95% 16–20, *p* = <.001). MRI revealed TAI lesions in 322 patients of MRIs performed (51%).

Multiple traumas were diagnosed in 1016/2153 (47%) with a mean age of 51 years (SD 22), compared to a mean age of 53 (SD 26) in those with isolated TBI (CI 95% 0.3–4, *p* = .03). The most frequent body regions affected in multitraumas were face (24%), thoracic (21%), extremities (14%) and cervical spine (12%), presented in Table 3. Spinal cord injury was present in 35/2153 (1.6%); 21 cervical and 14 thoracolumbar.

**Table 3:** Multiple trauma in TBI patients

	N (%) 2153 (100)
Multiple trauma (any)	1016 (47)
**Injury present and treatment given**^**a**^	
Face	517 (24)
*Conservative*	356 (17)
*Surgical*	161 (7)
Cervical fx^b^	263 (12)
*Conservative*	225 (10)
*Surgical*	38 (2)
Thoracic	460 (21)
*Conservative*	308 (14)
*Surgical*	152 (7)
Thoracolumbar fx	189 (9)
*Conservative*	145 (7)
*Surgical*	44 (2)
Abdominal	124 (6)
*Conservative*	87 (4)
*Surgical*	37 (2)
Pelvic fx	101(5)
*Conservative*	61 (3)
*Surgical*	40 (2)
Extremities fx	293 (14)
*Conservative*	109 (5)
*Surgical*	184 (9)

^a^ One patient may suffer from multiple injuries

^b^ fx: fracture

800/2153 (37%) were transported directly from scene of accident to OUH. Before transfer to OUH 816/2153 (38%) had primary assessment at local hospital, and 256/2153 (24%) had primary assessment at the Oslo Emergency Department. The OUH trauma team was activated in 1656/2153 (77%) patients with a mean age of 49 years (SD 24) compared to patients admitted without the trauma team who had a mean age of 61 years (SD 25) (CI 95% 9–14, *p* = <.001).

LOS in hospital and ICU are presented in Table 4. On average 9.0 TBI patients were hospitalized each day during the study period, and 5.4 of them were in the ICU. ICU admission was registered in 68%, these patients was slightly younger; mean age 50 years (SD 24), compared to patients not admitted ICU; 56 years (SD 25) (CI 95% 3–8, *p* = <.001). Of patients with severe TBI; 96% were admitted to ICU, with ICU-LOS median 3 days (IQR 2–8).

**Table 4:** Length of stay at hospital and ICU

Year	2015	2016	2017	2018	2019
	***N*** **= 383**	***N*** **= 433**	***N*** **= 404**	***N*** **= 481**	***N*** **= 452**
**Total LOS in days**					
Mean (SD)	8.2 (9)	8 (10)	8.5 (9.9)	6.5 (6.6)	7.2 (8.6)
Median (IQR)	5 (3–9)	4 (3–9)	4 (3–11)	4 (3–7)	4 (3–8)
Sum	3145	3473	3412	3115	3252
**ICU admission**	***N*** **= 281**	***N*** **= 306**	***N*** **= 283**	***N*** **= 299**	***N*** **= 287**
**LOS ICU in days**					
Mean (SD)	7 (8.6)	7.2 (9)	7.6 (10)	5.9 (7.6)	6.5 (8.7)
Median (IQR)	3 (2–9)	3 (2–8)	3 (2–10)	3 (2–6)	3 (2–6)
Sum	1952	2194	2142	1750	1874

*LOS* Length of stay, *ICU* intensive care unit, *SD* standard deviation, *IQR* interquartile range

**Fig. 4:**
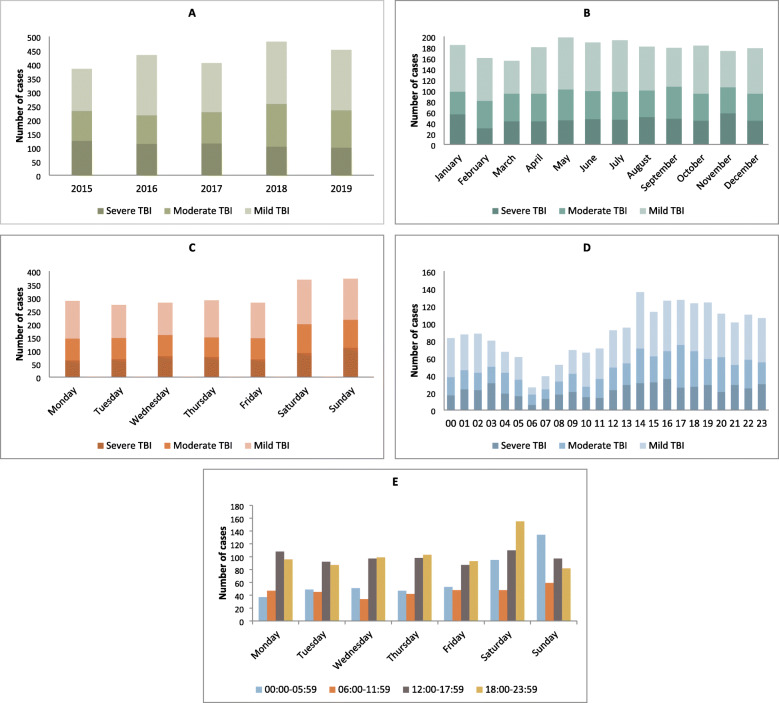
Distribution of admissions in period 2015–2019. TBI severity is based on HISS. **a**: Admission according to year. **b**: Admission according to month. **c**: Admission according to day of the week. **d**: Admission according to time in hours. **e**: Admission according to day of the week and time-intervals in hours

Patient load according to year, month, day of the week and time of day is presented in Fig. 4a-c. Mean annual number was 431 (SD 38, range 384–480) (Fig. 4a) and mean monthly number was 36 (SD 2, range 17–49) (Fig. 4b). Saturday and Sunday had the highest number of admissions; 34% (740/2153) (Fig. 4c). During the five-year period there was no significant variation in patient load per month (χ2 (44) =58, *p* = .08), distribution of TBI severity according to HISS per month (χ2 (22) =23, *p* = .4) or HISS per day of the week (χ2 (12) =9, *p* = .7). There was no significant variation of multitrauma between days of the week (χ2 (6) =9, *p* = .2).

The lowest admission rate during the day was in the morning at 06:00 (Fig. 4d). Admission rate gradually increased during morning and early afternoon and peaked around 14:00. The admittance continued at a high rate to midnight, declined a little after midnight and further declined after 04:00. During the whole week, significantly more TBI patients were admitted between 17:00 and 07:00 (χ2 (6) =15, *p* = .02). In weekends, significantly more patients were admitted during night, especially from midnight to 06:00 Saturday and Sunday (χ2 (3) =59, *p* = <.001) (Fig. 4e). Overall, 68% of the patients were admitted during evening, night or weekend.

**Fig. 5:**
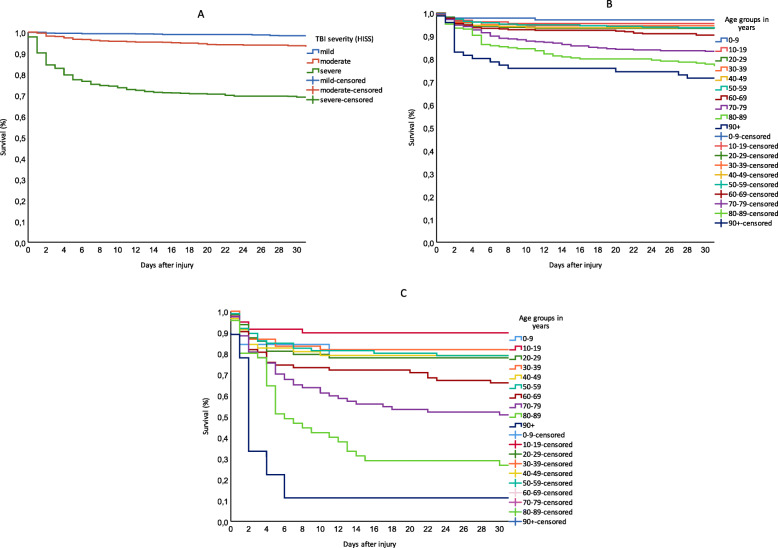
30-day survival between TBI severity and age groups. **a**: Survival between TBI severity. **b**: Survival between age groups. **c**: Survival for severe TBI between age groups

Survival estimates are presented in Fig. 5a-c. 30-day survival for mild, moderate and severe TBI was 98, 94 and 69%, respectively (*p* = <.001) (Fig. 5a). Figure 5b-c further shows how age is associated significantly with survival (*p* = <.001). For patients with severe TBI and < 60 years, most deaths occurred within 1 week after injury and 30-day survival was between 78 and 79%, except patients 10–19 years who had highest 30-days survival; 90%. There was a decline in survival from 60 year olds (66%), down to 11% in 90 year olds (Fig. 5c). The survival estimates include patients deemed unsalvageable upon arrival, and deaths occurring after discharge from OUH.

## Discussion

This is one of very few large-sample size studies that describes the TBI population admitted to trauma hospital from 2015 to 2019, in order to provide additional information and guide the hospital administration to adequately distribute its resources. The admission rate of TBI of all severities has been stable year-round since 2015. The largest patient group is from the late middle ages. Many of the patients are elderly with comorbidities and use of antithrombotic drugs is common. The majority of TBI patients were admitted when the hospital was outside ordinary working hours (Monday to Friday between 07.00 and 17:00), and 60% of the hospital stay was spent in the ICU. The TBI patients pose a significant burden to trauma hospitals due to high numbers, injury severity and complexity, need for ICU admittance, and need for all-hour presence of competent personnel.

Oslo TBI Registry – Neurosurgery had a higher mean age compared to previous epidemiological TBI studies (Peeters et al. 2015; Roozenbeek et al. 2013). However, results were in line with those from a recently published EU study from the same time period (2015 to 2017): median age 50 years (Steyerberg et al. 2019). Over the past decade, epidemiologic studies have described a shift from younger people typically injured in road crashes to increased incidence of fall injuries among the elderly in Western countries (Peeters et al. 2015; Pedersen et al. 2015; Koskinen and Alaranta 2008; Andelic et al. 2008; Roozenbeek et al. 2013; Peeters et al. 2017). Results from this study confirm this shift.

Patients with a pre-injury ASA-PS score of ≥ 3 were substantial (28%) compared to CENTER-TBI which was 11% (Steyerberg et al. 2019); the latter is similar to the general trauma population admitted to OUH (10%) (Sovik et al. 2014). The more silent comorbidity; antithrombotics, were used by 42% of our TBI patients > 50 years and this compares to other TBI studies, 33–47%, on the elderly population (Gaist et al. 2017; Julien et al. 2017; Narum et al. 2016; Lenell et al. 2019). In the total Norwegian population 18% ≥ 40 years use antithrombotic drugs (Berg et al. 2017) in line with previously mentioned EU study (Steyerberg et al. 2019).

Influence of alcohol at time of injury was common in all adult age groups and the overall proportion (27%) was in line with other European and local studies reporting proportions from 24 to 36% (Steyerberg et al. 2019; Nyholm et al. 2013; Harr et al. 2011; Owens et al. 2018; Andelic et al. 2010; Bakke et al. 2016; Bjarkø et al. 2019). In a previous study of TBI patients at OUH, 26% reported pre-injury substance abuse when screened by CAGE questionnaire (misuse: CAGE score ≥ 2), alcohol dominated (Andelic et al. 2010). Currently, no systematic assessments are made regarding substance use/dependence at admission to the hospital; hence the real proportion may be higher than our findings. The same clinical practice was found for approximately 10 years ago (Andelic et al. 2010), so the substance influence assessment at admission, preventive efforts and curative strategies need improvement.

Falls were the dominating trauma mechanism (55%) with a marked increase from age group 50 years in line with recent studies (Steyerberg et al. 2019). The late middle-aged (50–70 years) have recently been identified as a risk group, as they intersect with increasing comorbidity on one hand and keeping up physical and social activity on the other (Gale et al. 2018; Peeters et al. 2019). Thus, fall-preventing strategies ought to include this age-group as well. Overall, we found traffic accounted for a quarter of trauma mechanism. In Norway, systematic road safety efforts since the 1970s have resulted in a sharp reduction in road traffic deaths. However, the risk for injuries is higher for cyclists compared to drivers (Injuries in Norway, 2017). This is reflected in our study, the proportion of bicycle crashes was similar to MVC, and should be in focus of preventive efforts in the future.

In the entire TBI population it is estimated that 80–90% sustains mild TBI (Feigin et al. 2013; National Institute for Health and Care Excellence 2014). Our study population represents a small fraction (hospital admitted and abnormal CT) (see Fig. 1), yet we see close to half have a mild or minimal TBI based on HISS classification. A partial explanation can be use of antithrombotic drugs. First, the risk of hematoma/hemorrhage is increased (Gaist et al. 2017; Gulati et al. 2018). Second, according to Scandinavian guidelines (Unden et al. 2013) CT imaging is indicated in patients with mild TBI using anticoagulants or are ≥ 65 years and use antiplatelet therapy, thus identifying minor abnormalities on CT. Elderly who sustain a TBI more often have evidence of traumatic intracranial hemorrhage on CT (Andelic et al. 2008; Gardner et al. 2018). The most common intracranial CT abnormalities were tSAH, contusions and SDH, as in other studies (Steyerberg et al. 2019). Characteristics of younger patients were injuries of greater force, measured by trauma team activation, high-energy trauma and multitrauma.

High admittance rate to ICU suggests an aggressive treatment approach in initial phase in all ages. Nonetheless, survival in the older age groups was significantly lower. Higher mortality in older TBI patients is well-known from the literature, yet this group has often been excluded in clinical TBI studies (Gaastra et al. 2016). Controversy exists if old patients benefit from aggressive treatment. However, research has demonstrated improved outcome with aggressive treatment in elderly (Herou et al. 2015; Lenell et al. 2019; Bus et al. 2019; Whitmore et al. 2012). It is worth noting that although pre-injury comorbidity was high in older age groups, about 60% of patients 80–89 years still lived independently at home (Fig. 2b).

Studies from European trauma centers have reported a low threshold for ICU admission (Huijben et al. 2020; Volovici et al. 2019). Other studies question the necessity ICU admission or transfer to trauma center of low-risk TBI patients (Gates et al. 2017; Nishijima et al. 2011; Pruitt et al. 2017; Borczuk et al. 2019). A large proportion had a short LOS. Hence an issue for further investigation is whether we risk premature discharge for more severely injured patients who need specialized care, to be able to receive new patients. A part of this process would be a closer look at admissions to ICU and to trauma center for patients with mild TBI but not in need of intensive care, in light of current guidelines.

Studies on trauma populations describe trends towards increased admissions associated with warmer weather and summer months (northern temperate zone) (Kieffer et al. 2016; Ali and Willett 2015; Rising et al. 2006; Roislien et al. 2018; Bjarkø et al. 2019). However, our study did not identify significant variations between monthly admissions or TBI severity during the five-year period. On the contrary, at OUH TBI patients require a constant demand for acute treatment through the year. The admission rate does not just affect acute treatment, but includes the whole treatment chain. Consequently, there is a constant demand for qualified staff and beds in rehabilitation units as well.

We found a higher rate of admissions during the weekend, in accordance with other studies on trauma admissions (Kieffer et al. 2016; Stonko et al. 2018; Roislien et al. 2018; Bjarkø et al. 2019). The increased admission rate during afternoon, evening and at nighttime in weekends has also been found in other trauma centers (Stonko et al. 2018; Vaziri et al. 2007), both studies found penetrating trauma peaked around midnight – a few hours later than blunt trauma. Penetrating brain injuries is commonly caused by firearms, has high mortality, and has been reported to account for 12% of all TBI in the U.S. (Vakil and Singh 2017). Penetrating injury was an unusual trauma mechanism in our study (< 2%), thus not affecting time of admission.

Our results show that trauma activity is high when the hospital is mainly staffed for emergency care; particularly weekends are vulnerable in the execution of daily activities such as bedside visits. Knowing the high likelihood of interruptions and conflicts caused by new trauma; most of the planning and execution of elective inpatient treatment should be performed Monday to Friday between 07:00 and 17:00.

In the years after 2002, the trauma system at OUH was upgraded. As part of this process, neurosurgeons increased their all-hour presence and involvement in trauma patients. The result was increased survival in trauma patients, especially for patients with severe head/neck injury (Sovik et al. 2014). Our study supports the necessity for all-hour presence of neurosurgeons, as severely injured TBI patients arrive at all hours.

In high-income countries, observed short term survival for severe TBI have varied between 70 and 55% (Haller and Walder 2015), in accordance with our findings (69%). A large European multicenter study on TBI reported an in-hospital survival of 85% and 6-month survival of 79% in patients admitted to ICU (Steyerberg et al. 2019); this was patients with moderate-severe TBI (median GCS 9, median age 49, with abnormal neuroimaging in 90%). We found similar 30-day survival (78–90%) for severe TBI in patients < 60 years. Survival in the younger patients with severe TBI seems to have increased. However, survival in elderly patients decreased, and is in line with previous research (Gardner et al. 2018; Herou et al. 2015; Roe et al. 2013). This study illustrates the importance of including elderly with comorbidity in TBI research in order to increase the awareness of the implications this group has for clinical management and TBI outcome research. Further studies should emphasize on the associations between age, comorbidity and level of treatment, in order to ensure best possible outcome.

Some study limitations need to be addressed. The study was performed at a single Level 1 trauma center with neurosurgical service in southeast region of Norway. We did not calculate the incidence of hospital-admitted intracranial injuries in the region, as we did not have an overview of patients with minor intracranial injuries who were admitted and treated in general hospitals. Thus, the generalizability to patients treated in general hospitals, other geographical areas and countries with different health care organization is limited. Further, a well-known limitation of studies based on hospital medical records is missing data. Database coding errors can also be a limitation, but we have continuously searched and adjusted the database for inconsistency and coding errors. However, a large sample size, and prospective registration with no exclusion based on age, preinjury conditions or treatment are strengths of the study.

## Conclusion

In TBI patients admitted to OUH with abnormal neuroimaging, the largest group is the late middle-aged and pre-injury comorbidities are common. Fall is the major trauma mechanism. Patients are being admitted throughout the year, without seasonal differences. The case load is greatest when the hospital is mainly staffed for emergencies and care of critically ill inpatients, thus demanding an all-hour presence of competent personnel. Overall burden for the trauma hospital is considerable, shown by high numbers and high admission rates to the ICU. Given the national population composition by an aging population, we have no reason to believe the number of patients will decrease in near future.

## Data Availability

The datasets generated and/or analyzed during the current study are not publicly available due to the sensitivity of the material, but are available from the corresponding author on reasonable request.

## Abbreviations

ASA-PS: American Society of Anesthesiologists Physical Status Classification System
CT: Computed Tomography
EDH: Epidural hematoma
ER: Emergency room
GCS: Glasgow coma score
HISS: Head Injury Severity Score
ICU: Intensive care unit
IQR: Interquartile range
IVH: Intraventricular hemorrhage
LOS: Length of stay
MRI: Magnetic resonance imaging
MVC: Motor vehicle crash
OUH: Oslo University Hospital
SCI: Spinal cord injury
SD: Standard deviation
SDH: Acute subdural hematoma
TAI: Traumatic diffuse axonal injury
TBI: Traumatic Brain Injury
tSAH: Traumatic subarachnoid hemorrhage
